# MWCNTs-CTAB and HFs-Lac Nanocomposite-Modified Glassy Carbon Electrode for Rutin Determination

**DOI:** 10.3390/bios12080632

**Published:** 2022-08-11

**Authors:** Xin-Yan Song, Xin Meng, Bao-Lin Xiao, Yang-Yang Li, Xin-Xin Ma, Ali Akbar Moosavi-Movahedi, Jun Hong

**Affiliations:** 1 School of Life Sciences, Henan University, Kaifeng 475000, China; 2 Institute of Biochemistry and Biophysics, University of Tehran, Tehran 1417614418, Iran

**Keywords:** biosensor, laccase, hydroxyl fullerenes, multi-walled carbon nanotubes, rutin determination

## Abstract

Rutin is a flavonoid glycoside compound, which is mainly transported via the blood circulation system in the human body. The monitoring of the blood concentration of rutin is of great significance in many fields such as pharmacology and pharmacokinetics. In this work, a biosensor based on multi-walled carbon nanotubes (MWCNTs), cetyltrimethylammonium bromide (CTAB), hydroxyl fullerenes (HFs), and laccase (Lac) nanocomposite-modified glassy carbon electrodes was constructed. The modified materials were characterized with a transmission electron microscope (TEM), cyclic voltammograms (CV), and electrochemical impedance spectroscopy (EIS). CTAB is used to disperse MWCNTs and improve hydrophilicity and biocompatibility of MWCNTs, while the use of Lac can enhance the oxidation of catechol structure in rutin, thus significantly improving the sensitivity and selectivity of the modified electrode. Linear sweep voltammetry (LSV) studies showed that the determination linear ranges of rutin were 0.1 µmol L^−1^ to 2 µmol L^−1^ and 2 µmol L^−1^ to 11 µmol L^−1^, with the determination limits of 30 nmol L^−1^ and 95.5 nmol L^−1^, respectively. The proposed biosensor can be used to detect rutin tablets and serum samples with high recovery, which indicates a good accuracy of this method, and the results are consistent with those measured by the traditional ultra-high performance liquid chromatography (UHPLC) method. Hence, this biosensor has potential practical application value in rutin drug quality testing and clinical blood drug concentration monitoring.

## 1. Introduction

Rutin (3′,4′,5,7-tetrahydroxyflavone-3-rutinoside), also known as vitamin P and quercetin-3-o-rutinoside, mainly exists in *Sophora japonica*, *Calendula officinalis*, and *Amaranthus paniculatus* leaves. Rutin is a flavonoid glycoside compound with antioxidant, anti-inflammatory, antimicrobial, vascular protection, nerve protection, and other pharmacological activities [1,2,3,4,5,6]. Clinically, rutin has been used for the treatment of cardiovascular diseases, inflammatory diseases, lung cancer, [7,8,9], etc. In the human body, rutin is transported mainly via the blood circulation system, and can be absorbed directly through intestinal cells [10], so it is very important to monitor the blood concentration of rutin and to investigate the quality of the production of rutin. At present, the main methods of detecting rutin are high-pressure liquid chromatography (HPLC) [11], chemiluminescence (CL) [12], capillary electrophoresis (CE) [13], electrochemical method [14], etc. Among them, the electrochemical method has attracted extensive attention because of its portability of equipment, simple experiment process, low cost, high sensitivity, accuracy, and fast response [15,16]. A variety of materials have been used to construct electrochemical sensors for rutin determination, such as Ir NPs-IL-clay/PPO-Lac [17], AuNPs-CD-Lac [18], MWCNTs-IL-Gel [19], MIPIL/IL-GR [20], Fe_2.5_Cr_0.2_Ce_0.3_O_4_-rGO [21], etc.

Nanomaterials applied in electrochemical devices offer properties that are important in relation to the sensor interface at the molecular level [22]. Multi-walled carbon nanotubes (MWCNTs) have gained much attention because of their excellent properties, such as a thermal, electrical, mechanical, and current-carrying capability [23,24]. However, MWCNTs also have strong hydrophobicity and poor biocompatibility, therefore, in this work, cetyltrimethylammonium bromide (CTAB) was introduced to enhance MWCNTs’ hydrophilicity and biocompatibility and because it is beneficial for the subsequent modification of other materials [25]. Fullerenes (C_60_) have attracted attention in the field of sensors, due to their ability to enhance electrical conductivity, charge-transfer rates, and photophysical properties [26]. In addition, C_60_ has been used in strain sensors, gas sensors, electrochemical sensors, and optical sensors to detect organic molecules, biomolecules, small molecular compounds, metal ions, [27,28,29] etc. Hydroxyl fullerenes (HFs) are derived from a new material formed by the hydroxylation of C_60_; this structure can improve water solubility [30], and significantly protect reactive oxygen species oxidative damage to DNA and proteins [31]. Gao et al. reported HFs and glucose-oxidase-modified GCE to detect glucose [32]. Hence, using HF-immobilized enzyme to detect substrate can both improve the electrical signal and protect the enzyme. Furthermore, laccase (Lac) is a member of the blue multi-copper-oxidase family, and the substrate is phenolic compounds. In the presence of oxygen, phenolic compounds are oxidized to quinone compounds, and oxygen is reduced to water [33,34]. Several applications of Lac biosensor have been reported for the determination of catechol, hydroquinone, dopamine, chlorophene, and flavonoid compounds in food, the environment, and drugs [35,36,37,38,39,40,41,42].

In this work, the mixture of MWCNTs-CTAB was first modified on a glassy carbon electrode (GCE), and then the co-cultured mixture of HFs and Lac was added onto the modified electrode and, due to chitosan (Chi), had the ability to form stable films on surfaces [43]. Finally, a layer of Chi film was modified for electrode protection, so as to construct a biosensor capable of detecting rutin. Here, Lac was introduced to improve the selectivity and sensitivity of biosensors [44]. The reason for using MWCNTs and HFs was not only that they have good electrical conductivity and a high specific surface area [45], but also because HFs may protect the conformation and properties of proteins by forming complexes with proteins [46]. The materials used for the modification were characterized by transmission electron microscopy (TEM), cyclic voltammetry (CV), and electrochemical impedance spectroscopy (EIS). Then, the testing condition and other influencing factors, such as pH, determination time, proportion of modified material, and anti-interference ability and stability, were studied. Moreover, the practical application of this modified electrode was further evaluated in rutin quality testing and blood drug concentration monitoring.

## 2. Materials and Methods

### 2.1. Reagents and Materials

Lac (*Trametes versicolor*) and rutin were purchased from Yuanye Biology Co., Ltd. (Shanghai, China). MWCNTs were obtained from Shenzhen Nanotech Port Co., Ltd. (Shenzhen, China). HFs were obtained from Bucky (Houston, TX, USA), and CTAB was obtained from Shanghai chemical reagent procurement and supply station (Shanghai, China). Chi (about 100 kDa, 90% deacetylated) was obtained from Sangon Biotech Co., Ltd. (Shanghai, China). Sodium dihydrogen phosphate (NaH_2_PO_4_·2H_2_O) and disodium hydrogen phosphate (Na_2_HPO_4_·12H_2_O) were purchased from Sigma-Aldrich (Shanghai, China). Rutin solution was prepared with ethanol as the solvent, and 50 mol L^−1^ phosphate-buffered solution was used as a supporting electrolyte. All reagents were analytical-grade. All water used was prepared in an 18 MΩ cm ultrapure water machine in this study.

### 2.2. Apparatus and Measurements

All electrochemical measurements were carried out on a CHI660E electrochemical workstation (CH Instrument, Austin, TX, USA) at 25 °C. A GCE 3 mm in diameter, a Ag/AgCl-saturated electrode, and a platinum wire were used as the working electrode, reference electrode, and counter electrode, respectively. CV and linear sweep voltammetry (LSV) were carried out in 50 mmol L^−1^ pH 4 phosphate-buffered solution with a scan rate of 0.1 V s^−1^. Electrochemical impedance spectroscopy (EIS) was carried out in 0.1 M KCl solution containing 5 mmol L^−1^ [Fe(CN)_6_]^3−/4−^ in the frequency range from 10^2^ to 10^6^ Hz.

Currently, HPLC is generally used to determine rutin content in drugs and blood. Hence, HPLC was used as a comparative technique with the electrochemical method. Rutin tablets samples were detected using ultra-high-pressure liquid chromatograph (UHPLC) (1290 Infinity II, Agilent, Santa Clara, CA, USA) using the standard addition method. Separation was achieved using an Eclipse Plus C18 column (Agilent, Santa Clara, CA, USA) (2.7 μm, 4.6 × 100 mm); the mobile phase was methanol and water (65:35, *v*/*v*) at a flow rate of 0.2 mL min^−1^. The injection volume of the sample was 10 μL, and the UV detector wavelength was set at 360 nm [47].

TEM (JEM-2100, JEOL, Tokyo, Japan) images of MWCNTs, MWCNTs-CTAB, HFs, and HFs-Lac were taken at 200 KV [48]. The spectroscopic study of laccase catalyzing rutin was carried out on an ultraviolet and visible spectrophotometer (UV-vis spectrophotometer) (Evolution 220, Thermo, Shanghai, China).

### 2.3. Preparation of Modified Electrode

Ahead of modification, the surface of a GCE was mechanically polished with slurry of alumina powder with the particle sizes of 1, 0.3, and 0.05 μm, respectively. Then, the GCE was ultrasonicated in 75% ethanol and ultrapure water for 10 min. Afterwards, the GCE was placed in a drying tower.

Preparation of Chi solution: 50 mg of chitosan was added to 10 mL of ultra-pure water containing 1% acetic acid solution (*v*/*v* = 1:1), stirred until completely dissolved, and the pH was adjusted to 5–6 with the NaOH solution.

**Figure 1:**
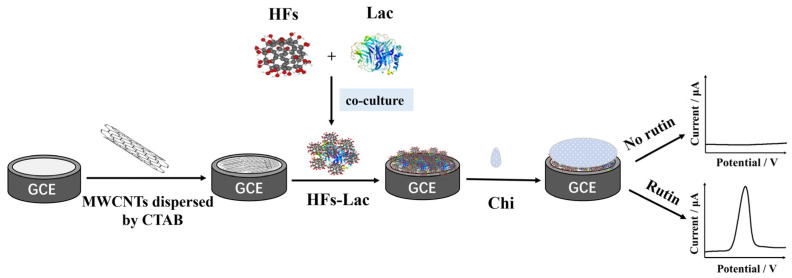
Preparation process of nanocomposite-modified GCE.

MWCNTs (2 mg mL^−1^) were ultrasonically dispersed in CTAB (2 mg mL^−1^), and 2 µL of the mixture of the MWCNTs-CTAB dispersive turbid liquid was dropped on the prepared GCE and dried at room temperature for 20 min. HFs (2 mg mL^−1^) and Lac (5 mg mL^−1^) were co-cultured at 4 °C for 1 h. Then, 2 μL of HFs-Lac co-culture solution was added on the electrode and dried at 4 °C overnight. At the end, in order to prevent the modified materials from falling off the electrode surface, 1.5 μL of Chi solution was dropped on the GCE, dried, and stored at 4 °C (Figure 1).

### 2.4. Preparation of Samples

Chi/HFs-Lac/MWCNTs-CTAB/GCE was used to determine rutin content in rutin tablets samples and serum samples. The preparation of rutin tablets samples was carried out as follows: rutin tablets were purchased from a pharmacy, the tablets were sonicated to dissolve in ethanol, and then the solution was centrifuged at 5000 r min^−1^ for 10 min after standing at room temperature for 1 h, and the supernatant was taken for electrochemical and UHPLC analyses. The preparation of rutin serum samples was performed as follows: Mouse blood was centrifuged at 3000 r min^−1^ for 10 min and the serum supernatant was obtained; then, the rutin solution was added to the serum and diluted for electrochemical analysis. Next, 1 mL of methanol was added to the serum sample of rutin (100 μL), then vortex-mixed for about 1 min, and left to stand at 4 °C for 10 min. After centrifugation at 12,000 r min^−1^, the supernatant was taken and blow-dried with nitrogen in water at 40 °C. Finally, the samples were redissolved in methanol for UHPLC analysis [49].

## 3. Results and Discussion

### 3.1. Characteristics of Modified Materials

**Figure 2:**
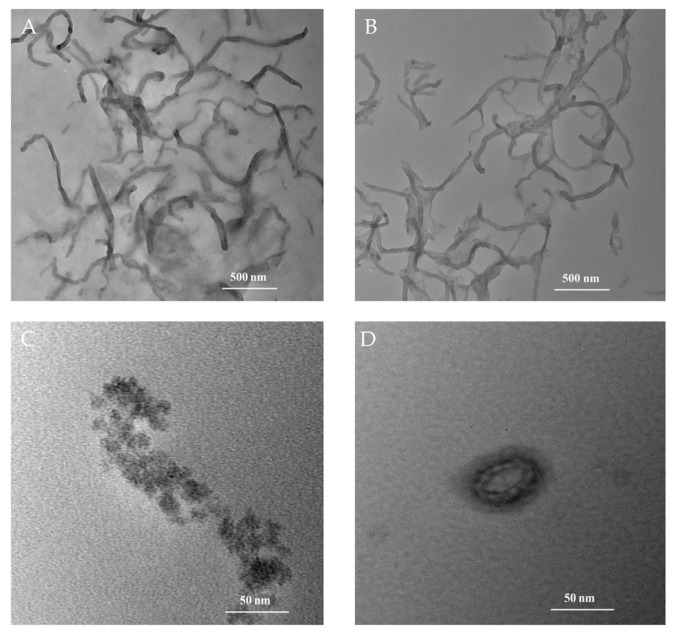
TEM images of MWCNTs (**A**), MWCNTs-CTAB (**B**), HFs (**C**), and HFs-Lac (**D**).

The TEM images of MWCNTs, MWCNTs-CTAB, HFs, and HFs-Lac are shown in Figure 2A–D, respectively. The MWCNTs mixed with CTAB in Figure 2B showed better dispersion than MWCNTs alone in Figure 2A. Figure 2C shows that the particle of HFs was about 15 nm, which might have been due to the dispersion of HFs in water and aggregation during dying. After HFs and Lac were cultured, Lac seemed to be wrapped by HFs, as shown in Figure 2D, which might have been conducive to the catalytic ability of Lac and the electrical conductivity of HFs.

**Figure 3:**
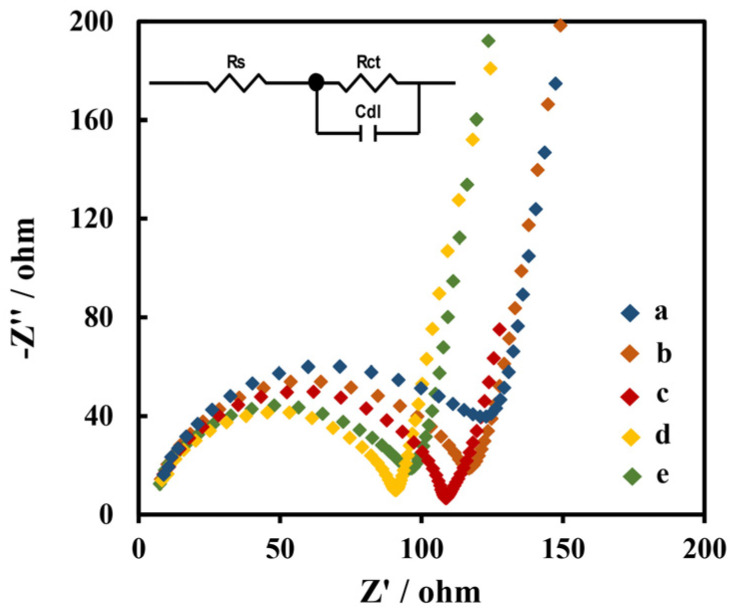
EIS of different nanometer-material-modified GCE: GCE (a); Chi/MWCNTs/GCE (b); Chi/MWCNTs-CTAB/GCE (c); Chi/HFs/MWCNTs-CTAB/GCE (d); and Chi/HFs-Lac/MWCNTs-CTAB/GCE (e), with a frequency range from 10^2^ to 10^6^ Hz in 0.1 M KCl solution containing 5 mmol L^−1^ [Fe(CN)_6_] ^3^^−/4^^−^. (Inset: Randle’s equivalent circuit. Rs: solution resistance; R_ct_: electron transfer resistance; C_dl_: double-layer capacitance).

The impedances of the electrodes modified with different materials were studied using the EIS method. The EIS diagrams of GCE, Chi/MWCNTs/GCE, Chi/MWCNTs-CTAB/GCE, Chi/HFs/MWCNTs-CTAB/GCE, and Chi/HFs-Lac/MWCNTs-CTAB/GCE are shown in Figure 3, respectively. The EIS curve consists of a semicircular part representing electron transfer resistance (R_ct_) and a liner part representing the adsorption process. The smaller semicircle diameter balances with the smaller R_ct_ value of the electrode, which means that there is a faster electron transfer rate [50]. The impedance data were fitted with Randle’s equivalent circuit in Figure 3 (inset). The R_ct_ of Chi/MWCNTs-CTAB/GCE (97 Ω) was lower than the R_ct_ of Chi/MWCNTs/GCE (117 Ω) and GCE (132 Ω), indicating that the MWCNTs had good conductivity, and the dispersion of CTAB might have helped to improve its effective electron transmission area. The R_ct_ of Chi/HFs/MWCNTs-CTAB/GCE decreased to 87 Ω; this is an indication that the HFs might have improved electron transfer on the electrode surface. However, the R_ct_ of Chi/HFs-Lac/MWCNTs-CTAB/GCE was increased to 95 Ω, which might have been due to Lac hindering the electron transport.

**Figure 4:**
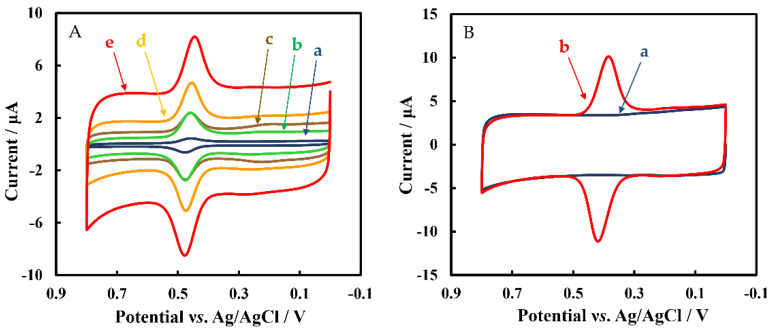
(**A**) CVs of different nanometer-material-modified GCEs in 50 mmol L^−1^ pH 4 phosphate-buffered solution containing 5 μmol L^−1^ rutin: GCE (a), Chi/MWCNTs/GCE (b), Chi/MWCNTs-CTAB/GCE (c), Chi/HFs/MWCNTs-CTAB (d), Chi/Lac/HFs/MWCNTs-CTAB (e). (**B**) CVs of Chi/Lac/HFs/MWCNTs-CTAB in 50 mmol L^−1^ pH 4 phosphate-buffered solution in the absence (a) and presence (b) of 5 μmol L^−1^ rutin.

Figure 4A shows the CVs of GCE, Chi/MWCNTs/GCE, Chi/MWCNTs-CTAB/GCE, Chi/HFs/MWCNTs-CTAB/GCE, and Chi/HFs-Lac/MWCNTs-CTAB/GCE in 5 μmol L^−1^ rutin, respectively. The bare GCE (curve a) had only a weak oxygen reduction peak, while the Chi/MWCNTs (curve c) and Chi/MWCNTs-CTAB (curve b) modified GCEs showed obvious redox peaks. After modification of the Chi/MWCNTs-CTAB/GCE with HFs (curve d), strong reduction signals were displayed at 0.473 V and 0.455 V, respectively. The Chi/HFs-Lac/MWCNTs-CTAB/GCE (curve e) showed the strongest oxidation peak and reduction peak at 0.478 V and 0.445 V, respectively, with a difference potential (ΔE) of 0.033 V (ΔE > 59/*n* mV, *n* = 2). The anodic peak current (I_pa_) and cathodic peak current (I_pc_) were 4.69 μA, 4.34 μA, respectively, with a peak current ratio (I_pa_/I_pc_) of about 1. Therefore, the Chi/HFs-Lac/MWCNTs-CTAB/GCE electrochemical process was quasi-reversible. Compared with Chi/MWCNTs/GCE, Chi/MWCNTs-CTAB/GCE had a larger electroactive surface area. At the same time, the HFs-Lac structure was more conducive to improving the catalytic performance of Lac, which made up for its low electrical conductivity to a certain extent. Figure 4B shows CVs of Chi/HFs-Lac/MWCNTs-CTAB/GCE in the absence (curve a) and presence (curve b) of 5 μmol L^−1^ rutin in phosphate-buffered solution. The results further indicate that the redox peaks were caused by rutin.

### 3.2. Effects of Scan Rates

**Figure 5:**
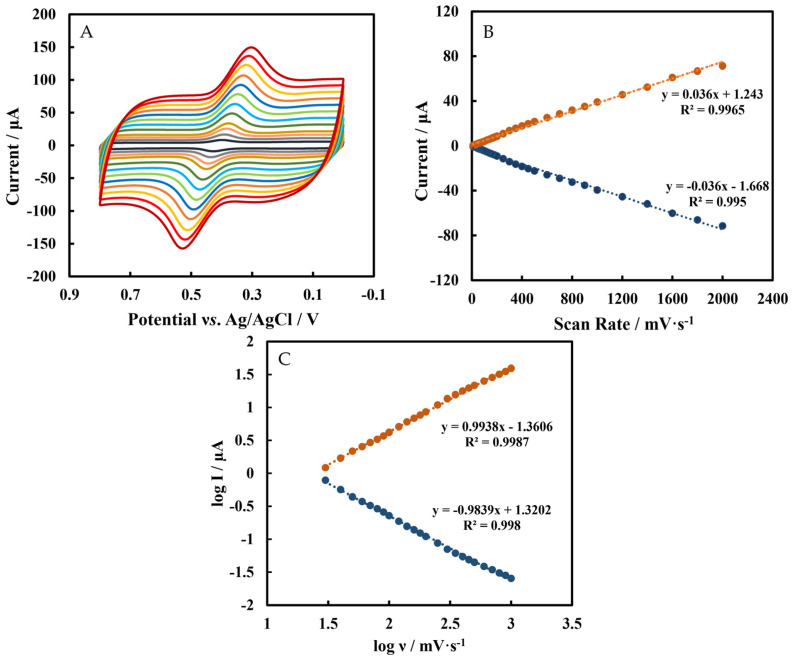
(**A**) CVs of Chi/HFs-Lac/MWCNTs-CTAB/GCE in pH 4 50 mmol L^−1^ phosphate−buffered solution containing 5 μmol L^−1^ rutin at scan rates (from inner to outer) of: 0.1, 0.2, 0.3, …, 1.4, 1.6, 1.8, 2 V s^−1^ (**B**) The relationship between scan rates (0.01 V s^−1^–2 V s^−1^) and peak currents. (**C**) The relationship between log *ν* and log I.

CVs of Chi/HFs-Lac/MWCNTs-CTAB/GCE in 5 µmol L^−1^ rutin at different scan rates (*ν*) are shown in Figure 5A, and the relationship between scan rates (0.01 V s^−1^–2 V s^−1^) and peak current is shown in Figure 5B. The regression equations were I_pc_ = 0.036 *ν* + 1.243 (R^2^ = 0.9965) and I_pa_ = −0.036 *ν* − 1.668 (R^2^ = 0.995). In Figure 5C, the absolute slopes value of log I vs. log *ν* were close to 1, so these indicate that the electrochemical behavior of rutin on the electrode surface is an adsorption-controlled process [16], which is the same as in other reports [51,52]. This phenomenon suggests that the determination of rutin should start from a low concentration, or the adsorbed rutin in the blank phosphate-buffered solution should be removed after detecting a high concentration of rutin. In addition, according to Equation (1) and the slope of the peak current (*I_p_*) versus the scan rate (*ν*) (Figure 5B), the number (*n*) of electron transfer in the electrode reaction was 2, which is same result as the previous report [53,54].

(1) $$I_{p}=\frac{nFQ\nu }{4RT}$$

Here, *F*, *Q*, *R*, and *T* represent the Faraday constant, the quantity of the electric charge, and the gas constant and temperature, respectively.

### 3.3. Effects of pH Values

**Figure 6:**
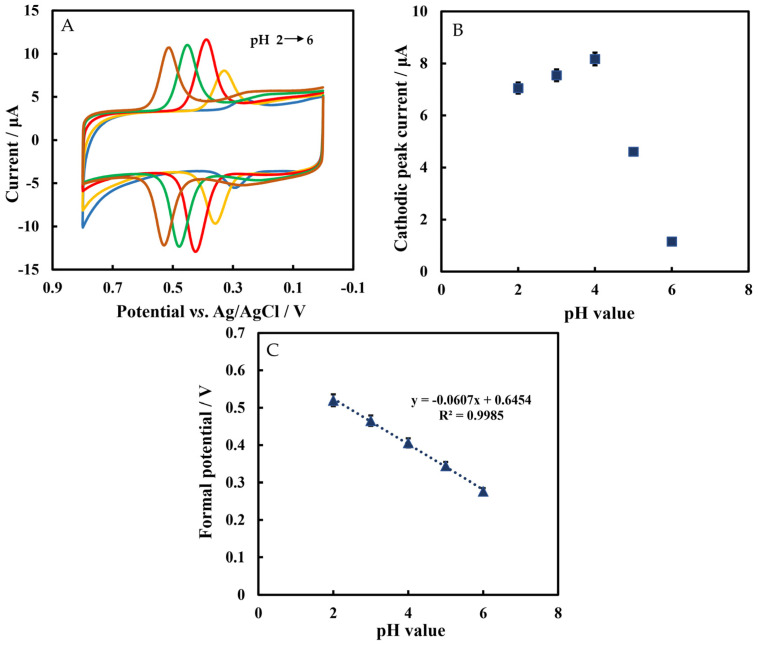
(**A**) The CVs of Chi/HFs-Lac/MWCNTs-CTAB/GCE in 50 mmol L^−1^ phosphate-buffered solution at different pH values containing 5 μmol L^−1^ rutin. (**B**) Plot of cathodic peak current versus pH value. (**C**) Plot of formal potential versus pH value.

Figure 6A shows the CVs of Chi/HFs-Lac/MWCNTs-CTAB/GCE in phosphate-buffered solution containing 5 μmol L^−1^ rutin at different pH values. Figure 6B shows that the cathodic peak current increased with a pH value from 2 to 4, but the current decreased with a pH value higher than 4. It was also reported that the optimum pH range of Lac catalytic activity was 3–4 [55]; therefore, the pH 4 phosphate-buffered solution was selected as the test solution. Furthermore, the formal potential was linearly related to pH with the equation of E°′ = −0.0607 pH + 0.645 (R^2^ = 0.998). The slope value was −60.7 mV pH^−1^, which was close to the Nernst’s value of 59.2 mV pH^−1^, indicating that the number of electrons and protons was the same in this reaction (Figure 6C). The pKa value of rutin is 6.17 ± 0.4, while the pH values studied in this work were from 2 to 6, so rutin was still protonated.

### 3.4. Optimization of Experimental Conditions

**Figure 7:**
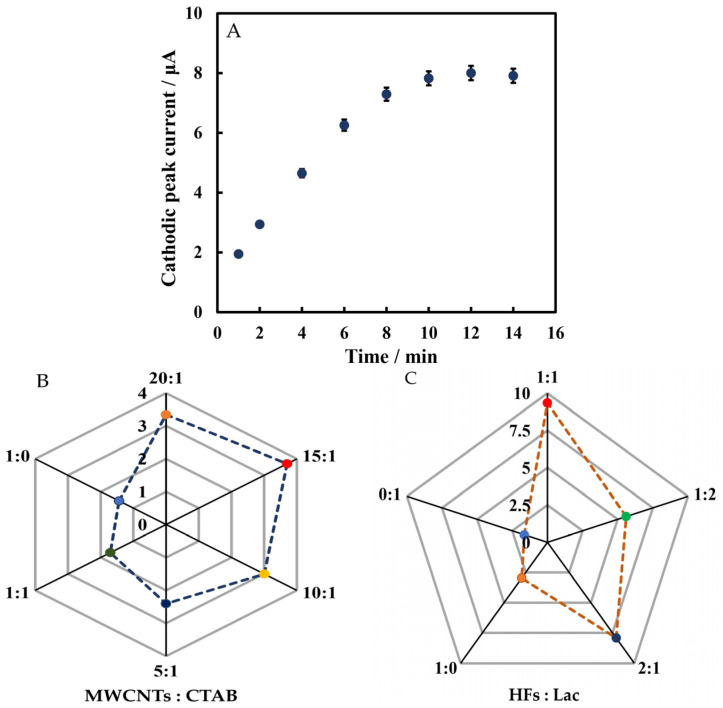
LSVs versus determination time (**A**), the optimal ratios of MWCNTs to CTAB (**B**) and HFs to Lac (**C**) for the the modified electrode in 50 mmol L^−1^ pH 4 phosphate-buffered solution containing 5 µmol L^−1^ rutin.

In order to optimize the performance of Chi/HFs-Lac/MWCNTs-CTAB/GCE, the determination time and ratio of modified materials were investigated. From 1 to 10 min, the cathodic peak current increased with time, and after 10 min, the current was basically stable (Figure 7A). Therefore, rutin was tested at 10 min. The optimal ratios of MWCNTs to CTAB and HFs to Lac were studied. As seen in Figure 7B,C, the cathodic peak signal was strongest when the ratio of MWCNTs to CTAB was 15:1 (*v*/*v*), and that of HFs to Lac was 1:1 (*v*/*v*). Thus, these optimal ratios were used for electrode modification.

### 3.5. Calibration Plot of Rutin

**Figure 8:**
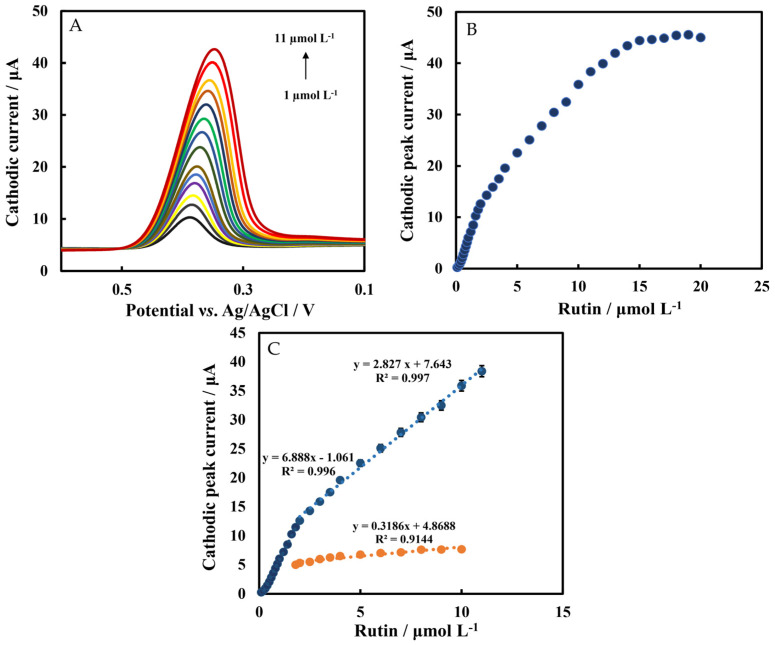
(**A**) LSVs of Chi/HFs-Lac/MWCNTs-CTAB/GCE at different concentrations of rutin. (**B**) Plot of cathodic peak current versus rutin concentrations. (**C**) Cathodic peak current versus rutin concentrations of Chi/HFs-Lac/MWCNTs-CTAB/GCE (blue line) and Chi/HFs/MWCNTs-CTAB/GCE (orange line). The experiments were carried out in 50 mmol L^−1^ pH 4 phosphate-buffered solution at a scan rate of 0.1 V s^−1^.

The LSV method was used to determine rutin under optimal conditions (Figure 8A). The plot of cathodic peak current versus rutin in the concentration range of 0.05 μmol L^−1^–20 μmol L^−1^ is shown in Figure 8B. The linear range of Chi/HFs-Lac/MWCNTs-CTAB/GCE response to rutin was divided into two parts: 0.1 μmol L^−1^–2 μmol L^−1^ and 2 μmol L^−1^ to 11 μmol L^−1^, with regression equations of I (μA) = 6.888C_Rutin_ (μmol L^−1^) − 1.061 and I (μA) = 2.827C_Rutin_ (μmol L^−1^) + 7.643, respectively (Figure 8C), and the limit of detection(LOD) was 30 nmol L^−1^ and 95.5 nmol L^−1^ (Equation (2), which is better than that previously reported [17,18]). The sensitivities of the biosensor were 6.88 μA μM^−1^ and 2.82 μA μM^−1^, respectively, which were better than those without Lac modification, which demonstrated the catalytic activity of the Lac-modified electrode. Table 1 shows a comparison between this work and the performance of earlier reported rutin sensors. It can be seen that the improved modified biosensor in this work had good performance.

LOD = 3 S_0_/S (2)

Here, S_0_ and S represent the standard deviation measured under blank solution and the slope of the linear range curve, respectively.

**Table 1:** Comparison of the performances of electrochemical sensors for determination of Rutin.

Working Electrode	Liner Range (μmol L^−1^)	LOD (nmol L^−1^)	Sensitivity (μA μM^−1^ )	Reference
Lac/GCE	0.1–0.7 0.7–1.3	--	5.85 2.47	[14]
Ir NPs-IL-clay/PPO-Lac/CPE	0.0917–3.1	30.9	4.26	[17]
AuNPs-CD-Lac/CPE	0.3–2.97	170	4.51	[18]
MWCNTs-IL-Gel/GCE	0.072–6	20	3.666	[19]
MIPIL/IL-GR	0.03–1	10	0.183	[20]
Fe_2.5_Cr_0.2_Ce_0.3_O_4_-rGO/GCE	0.075–12	52	2.988	[21]
Chi-Lac-TPP-CPE	0.599–3.92 5.82–13.1	0.0623 0.712	3.195 0.77	[34]
MWCNTs-CD-Fe_3_O_4_/GCE	0.02–10	16.4	1.24	[51]
Gr/GNR	0.032–0.1	7.86	-	[56]
MIP/AuNPs-MoS_2_-GN/GCE	0.01–45	4	1.146	[57]
Chi/HFs-Lac/MWCNTs-CTAB/GCE	0.1–2 2–11	30 95.5	6.888 2.827	This work

Lac: laccase; Ir NPs: iridium nanoparticles; IL: ionic liquid; PPO: polyphenol oxidase; CPE: carbon paste electrode; AuNPs: gold nanoparticles; CD: β-cyclodextrin; MIP: molecularly imprinted polymer; rGO: reduced graphene oxide; TPP: tripolyphosphate; Fe_3_O_4_ NPs: Fe_3_O_4_ nanoparticles; Gr: graphite; GNR: graphene nanoribbon; GN: graphene.

### 3.6. Anti-Interference Ability and Stability of Biosensor

**Figure 9:**
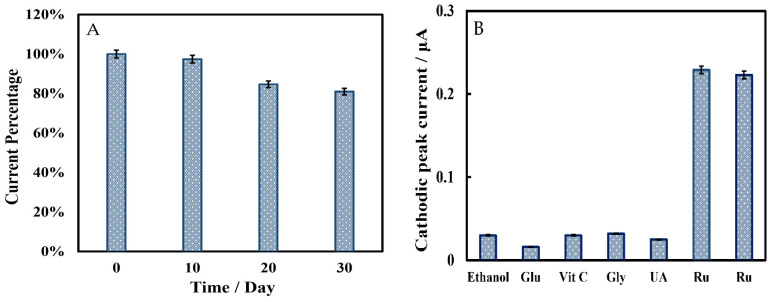
(**A**) Storage stability of Chi/HFs-Lac/MWCNTs-CTAB/GCE in 50 mmol L^−1^ pH 4 phosphate-buffered solution containing 5 μmol L^−1^ rutin. (The biosensor has stored at 4 °C for 0, 10, 20, 30 days.) (**B**) Anti-interference ability of Chi/HFs-Lac/MWCNTs-CTAB/GCE in 50 mmol L^−1^ pH 4 phosphate-buffered solution containing 250 μmol L^−1^ different interfering substance (ethanol, Glu, Vit C, Gly, UA) and 0.5 μmol L^−1^ rutin.

The storage stability of the biosensor was evaluated using the LSV method in phosphate-buffered solution containing 5 μmol L^−1^ rutin (Figure 9A). The cathodic peak current showed little change when the biosensor was stored at 4 °C for 10 days. After 20 and 30 days of storage, the peak signal decreased by 15% and 19%, respectively. The anti-interference ability of the biosensor was studied by observing whether the electroactive species changed the cathodic peak current using the LSV method. In Figure 9B, interfering substances in 500-fold concentrations of rutin (250 µmol L^−1^), including ethanol, glucose (Glu), ascorbic acid (Vit C), glycine (Gly), and uric acid (UA), elicited only weak current responses. Both additions of 0.5 μmol L^−1^ rutin caused a strong current signal and a cathodic peak appeared, so this biosensor had a great anti-interference performance. Due to the different concentrations of interfering substances and rutin samples, the cathodic peak current could not accurately describe the anti-interference capability of this biosensor. Here, we introduced an interference factor (IF) (Equation (3)) to more intuitionally investigate the anti-interference ability of the biosensor. The lower the IF value, the stronger the anti-interference ability of the biosensor. The results are shown in Table 2.

IF = S _interference_/S_rutin_ × 100% (3)

Here, S_interference_ and S_rutin_ represent the sensitivity of interfering substances and sensitivity of rutin, respectively.

**Table 2:** The IF values of different interfering substances.

Interfering Substance	Ethanol	Glu	Vit C	Gly	UA
IF (%)	0.03	0.01	0.03	0.03	0.02

**Figure 10:**
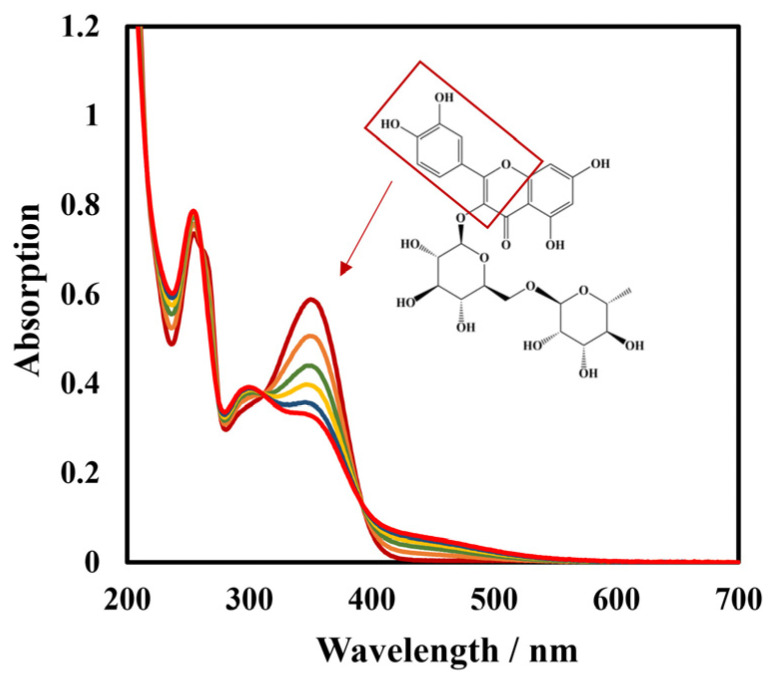
UV-vis spectrogram of continuous scanning Lac and rutin reaction system in 50 mmol L^−1^ pH 4 phosphate-buffered solution from 200 nm to 700 nm.

**Figure 11:**
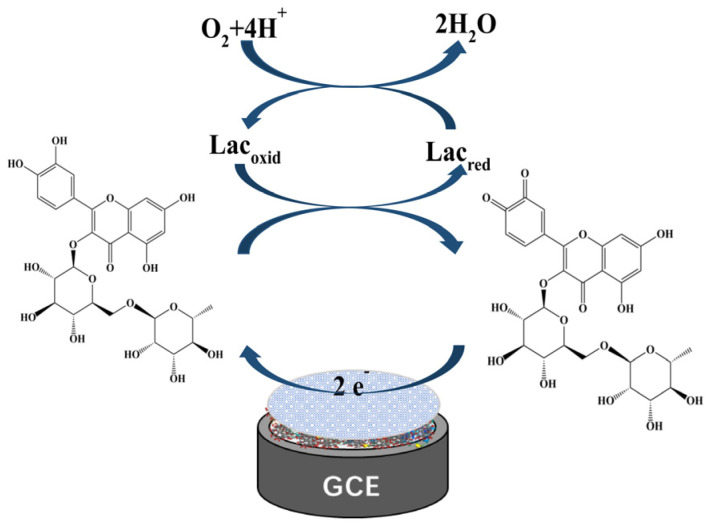
Schematic diagram of rutin catalyzed by Lac on the biosensor surface.

To further verify the specificity of this biosensor, spectra of laccase-catalyzed rutin were studied. Continuous spectral scans from 200 nm to 700 nm of Lac and the rutin reaction system using UV-vis spectrophotometer are shown in Figure 10. The absorption bands at 354 nm and 254 nm are cinnamoyl chromophore and benzoyl chromophore of rutin, respectively [58]. It can be seen that the absorption peak of the cinnamoyl structure at 354 nm decreased with the reaction time, indicating that the catechol of this structure was oxidized. Lac catalyzed the oxidation of rutin to the corresponding o-quinone with the concomitant reduction of molecular oxygen to water; the corresponding o-quinone served as the two electrons’ acceptor, and was reduced back to rutin on the surface of the biosensor (Figure 11).

### 3.7. Determination of Rutin in Tablets and Serum

Rutin tablets and serum samples were determined using the proposed biosensor. The results of electrochemistry and UHPLC methods used to determine rutin tablets samples with different concentrations are shown in Table 3. In addition, the electrochemistry method was used to determine different concentrations of rutin in serum samples. The results show that the biosensor had a good recovery (Table 4). Statistical analysis showed that the f-test value was lower than the critical value of F (F_critical_ = 5.05), while the t-test value was lower than the critical value of t (t_critical_ = 2.57), indicating that the biosensor is comparable to the traditional UHPLC method. Therefore, the improved modified biosensor has potential practical application value in rutin drug quality determination and clinical blood drug concentration monitoring.

## 4. Conclusions

The Chi/HFs-Lac/MWCNTs-CTAB/GCE biosensor was prepared for the determination of rutin with a wide determination linear range, low detection limit, high accuracy, good anti-interference ability, and strong stability. The biosensor had the same accuracy as traditional methods such as UHPLC in determining rutin and can be applied to the field of drug quality determination. Moreover, the biosensor had a good recovery for detecting rutin serum samples using the standard addition method; so the biosensor has potential practical application value in blood drug concentration monitoring.

## Figures and Tables

**Table 3:** Rutin tablet sample determinations using UHPLC and electrochemical methods.

Concentration (μmol L^−1^)	Biosensor (μmol L^−1^)	Recovery (%)	RSD (%)	UHPLC (μmol L^−1^)	Recovery (%)
1	0.98	98	1.14	0.98	98
5	4.84	96.8	2.76	4.91	98.2
10	9.86	98.6	2.37	9.83	98.3

F = 1.01 (F_critical_ = 5.05); t = 2.19 (t_critical_ = 2.57).

**Table 4:** Rutin serum sample determinations using electrochemical method.

Concentration (μmol L^−1^)	Biosensor (μmol L^−1^)	Recovery (%)	RSD (%)	UHPLC (μmol L^−1^)	Recovery (%)
0.5	0.472	94.4	2.69	0.515	103
1	1.03	103	2.14	1.15	115
2.5	2.44	97.7	1.88	2.64	105

F = 1.48 (F_critical_ = 5.05); t = 1.6 (t_critical_ = 2.57).

## Data Availability

Not applicable.

## References

[1] Zhao B.-Y., Xu P., Yang F.-X., Wu H., Zong M.-H., Lou W.-Y. (2015). Biocompatible Deep Eutectic Solvents Based on Choline Chloride: Characterization and Application to the Extraction of Rutin from Sophora japonica. ACS Sustain. Chem. Eng..

[2] Loescher C.M., Morton D.W., Razic S., Agatonovic-Kustrin S. (2014). High performance thin layer chromatography (HPTLC) and high performance liquid chromatography (HPLC) for the qualitative and quantitative analysis of Calendula officinalis—Advantages and limitations. J. Pharm. Biomed. Anal..

[3] Kraujalis P., Venskutonis P.R., Ibáñez E., Herrero M. (2015). Optimization of rutin isolation from Amaranthus paniculatus leaves by high pressure extraction and fractionation techniques. J. Supercrit. Fluids.

[4] Cushnie T.P., Lamb A.J. (2005). Antimicrobial activity of flavonoids. Int. J. Antimicrob. Agents.

[5] Ganeshpurkar A., Saluja A.K. (2017). The Pharmacological Potential of Rutin. Saudi Pharm. J..

[6] Chua L.S. (2013). A review on plant-based rutin extraction methods and its pharmacological activities. J. Ethnopharmacol..

[7] Kalita B., Das M.K. (2018). Rutin-phospholipid complex in polymer matrix for long-term delivery of rutin via skin for the treatment of inflammatory diseases. Artif. Cells Nanomed. Biotechnol..

[8] Paudel K.R., Wadhwa R., Tew X.N., Lau N.J.X., Madheswaran T., Panneerselvam J., Zeeshan F., Kumar P., Gupta G., Anand K. (2021). Rutin loaded liquid crystalline nanoparticles inhibit non-small cell lung cancer proliferation and migration in vitro. Life Sci..

[9] Filipsky T., Riha M., Haskova P., Pilarova V., Novakova L., Semecky V., Vavrova J., Holeckova M., Palicka V., Simunek T. (2017). Intravenous rutin in rat exacerbates isoprenaline-induced cardiotoxicity likely due to intracellular oxidative stress. Redox Rep..

[10] Chen I.L., Tsai Y.J., Huang C.M., Tsai T.H. (2010). Lymphatic absorption of quercetin and rutin in rat and their pharmacokinetics in systemic plasma. J. Agric. Food Chem..

[11] Mohamed S., Youssef A., Issa M., Abdel Salam H., El-Ansary A. (2020). Validated HPLC Method for Quantitative Analysis of Gallic Acid and Rutin in Leaves of Moringa Oleifera Grown in Egypt. Egypt. J. Chem..

[12] Tan H., Zhao Y., Xu X., Sun Y., Li Y., Du J. (2019). A covalent triazine framework as an oxidase mimetic in the luminol chemiluminescence system: Application to the determination of the antioxidant rutin. Microchim. Acta.

[13] Memon A.F., Solangi A.R., Memon S.Q., Mallah A., Memon N., Memon A.A. (2016). Simultaneous Determination of Quercetin, Rutin, Naringin, and Naringenin in Different Fruits by Capillary Zone Electrophoresis. Food Anal. Methods.

[14] Malinowski S., Wardak C., Jaroszynska-Wolinska J., Herbert P.A.F., Panek R. (2018). Cold Plasma as an Innovative Construction Method of Voltammetric Biosensor Based on Laccase. Sensors.

[15] Yang X., Long J., Sun D. (2016). Highly-sensitive Electrochemical Determination of Rutin Using NMP-Exfoliated Graphene Nanosheets-Modified Electrode. Electroanalysis.

[16] Antoniazzi C., de Lima C.A., Marangoni R., de Castro E.G., Santana E.R., Spinelli A. (2020). Molybdenum trioxide incorporated in a carbon paste as a sensitive device for bisphenol A monitoring. Microchem. J..

[17] Zapp E., Brondani D., Vieira I.C., Dupont J., Scheeren C.W. (2010). Bioelectroanalytical Determination of Rutin Based on bi-Enzymatic Sensor Containing Iridium Nanoparticles in Ionic Liquid Phase Supported in Clay. Electroanalysis.

[18] Brugnerotto P., Silva T.R., Brondani D., Zapp E., Vieira I.C. (2017). Gold Nanoparticles Stabilized in β-Cyclodextrin and Decorated with Laccase Applied in the Construction of a Biosensor for Rutin. Electroanalysis.

[19] Liu X., Li L., Zhao X., Lu X. (2010). Electrochemical behavior of rutin on a multi-walled carbon nanotube and ionic liquid composite film modified electrode. Colloids Surf. B.

[20] Lu Y., Hu J., Zeng Y., Zhu Y., Wang H., Lei X., Huang S., Guo L., Li L. (2020). Electrochemical determination of rutin based on molecularly imprinted poly (ionic liquid) with ionic liquid-graphene as a sensitive element. Sens. Actuators B Chem..

[21] Şenocak A., Korkmaz E., Khataee A., Demirbas E. (2022). A facile and synergetic strategy for electrochemical sensing of rutin antioxidant by Ce–Cr doped magnetite@rGO. Mater. Chem. Phys..

[22] Zapp E., Westphal E., Gallardo H., de Souza B., Cruz Vieira I. (2014). Liquid crystal and gold nanoparticles applied to electrochemical immunosensor for cardiac biomarker. Biosens. Bioelectron..

[23] Beitollahi H., Movahedifar F., Tajik S., Jahani S. (2018). A Review on the Effects of Introducing CNTs in the Modification Process of Electrochemical Sensors. Electroanalysis.

[24] Niu N.-N., Zhao W.-J., Xiao B.-L., Liang Y.-C., Meng X., Song X.-Y., Li D., Hong J., Moosavi-Movahedi A.A. (2021). Quench treatment cytochrome c: Transformation from a classical redox protein to a peroxidase like enzyme. J. Iran. Chem. Soc..

[25] Yang Y.J., Guo L., Zhang W. (2016). The electropolymerization of CTAB on glassy carbon electrode for simultaneous determination of dopamine, uric acid, tryptophan and theophylline. J. Electroanal. Chem..

[26] Shetti N.P., Mishra A., Basu S., Aminabhavi T.M. (2021). Versatile fullerenes as sensor materials. Mater. Today Chem..

[27] Brahman P.K., Pandey N., Topkaya S.N., Singhai R. (2015). Fullerene-C_60_-MWCNT composite film based ultrasensitive electrochemical sensing platform for the trace analysis of pyruvic acid in biological fluids. Talanta.

[28] Keshtkar S., Rashidi A., Kooti M., Askarieh M., Pourhashem S., Ghasemy E., Izadi N. (2018). A novel highly sensitive and selective H_2_S gas sensor at low temperatures based on SnO_2_ quantum dots-C_60_ nanohybrid: Experimental and theory study. Talanta.

[29] Palanisamy S., Thirumalraj B., Chen S.M., Ali M.A., Al-Hemaid F.M. (2015). Palladium nanoparticles decorated on activated fullerene modified screen printed carbon electrode for enhanced electrochemical sensing of dopamine. J. Colloid Interface Sci..

[30] Wang Z., Wang S., Lu Z., Gao X. (2015). Syntheses, Structures and Antioxidant Activities of Fullerenols: Knowledge Learned at the Atomistic Level. J. Clust. Sci..

[31] Cai X., Jia H., Liu Z., Hou B., Luo C., Feng Z., Li W., Liu J. (2008). Polyhydroxylated fullerene derivative C_60_(OH)_24_ prevents mitochondrial dysfunction and oxidative damage in an MPP^+^-induced cellular model of Parkinson’s disease. J. Neurosci. Res..

[32] Gao Y.F., Yang T., Yang X.L., Zhang Y.S., Xiao B.L., Hong J., Sheibani N., Ghourchian H., Hong T., Moosavi-Movahedi A.A. (2014). Direct electrochemistry of glucose oxidase and glucose biosensing on a hydroxyl fullerenes modified glassy carbon electrode. Biosens. Bioelectron..

[33] Rodríguez-Delgado M.M., Alemán-Nava G.S., Rodríguez-Delgado J.M., Dieck-Assad G., Martínez-Chapa S.O., Barceló D., Parra R. (2015). Laccase-based biosensors for detection of phenolic compounds. TrAC Trends Anal. Chem..

[34] Fernandes S.C., de Oliveira I.R.W.Z., Fatibello-Filho O., Spinelli A., Vieira I.C. (2008). Biosensor based on laccase immobilized on microspheres of chitosan crosslinked with tripolyphosphate. Sens. Actuators B-Chem..

[35] Liu L., Anwar S., Ding H., Xu M., Yin Q., Xiao Y., Yang X., Yan M., Bi H. (2019). Electrochemical sensor based on F,N-doped carbon dots decorated laccase for detection of catechol. J. Electroanal. Chem..

[36] Bilir K., Weil M.-T., Lochead J., Kök F.N., Werner T. (2016). Construction of an oxygen detection-based optic laccase biosensor for polyphenolic compound detection. Turk. J. Biol..

[37] Ferry Y., Leech D. (2005). Amperometric Detection of Catecholamine Neurotransmitters Using Electrocatalytic Substrate Recycling at a Laccase Electrode. Electroanalysis.

[38] Lou C., Jing T., Zhou J., Tian J., Zheng Y., Wang C., Zhao Z., Lin J., Liu H., Zhao C. (2020). Laccase immobilized polyaniline/magnetic graphene composite electrode for detecting hydroquinone. Int. J. Biol. Macromol..

[39] Zhang Y., Li X., Li D., Wei Q. (2020). A laccase based biosensor on AuNPs-MoS_2_ modified glassy carbon electrode for catechol detection. Colloids Surf. B.

[40] Franzoi A.C., Vieira I.C., Dupont J., Scheeren C.W., de Oliveira L.F. (2009). Biosensor for luteolin based on silver or gold nanoparticles in ionic liquid and laccase immobilized in chitosan modified with cyanuric chloride. Analyst.

[41] Boubezari I., Bessueille F., Bonhomme A., Raimondi G., Zazoua A., Errachid A., Jaffrezic-Renault N. (2020). Laccase-Based Biosensor Encapsulated in a Galactomannan-Chitosan Composite for the Evaluation of Phenolic Compounds. Biosensors.

[42] Quintanilla-Villanueva G.E., Luna-Moreno D., Blanco-Gamez E.A., Rodriguez-Delgado J.M., Villarreal-Chiu J.F., Rodriguez-Delgado M.M. (2021). A Novel Enzyme-Based SPR Strategy for Detection of the Antimicrobial Agent Chlorophene. Biosensors.

[43] Santana E.R., Martins E.C., Spinelli A. (2021). Electrode modified with nitrogen-doped graphene quantum dots supported in chitosan for triclocarban monitoring. Microchem. J..

[44] Alvarado-Ramirez L., Rostro-Alanis M., Rodriguez-Rodriguez J., Sosa-Hernandez J.E., Melchor-Martinez E.M., Iqbal H.M.N., Parra-Saldivar R. (2021). Enzyme (Single and Multiple) and Nanozyme Biosensors: Recent Developments and Their Novel Applications in the Water-Food-Health Nexus. Biosensors.

[45] Sherigara B.S., Kutner W., D’Souza F. (2003). Electrocatalytic Properties and Sensor Applications of Fullerenes and Carbon Nanotubes. Electroanalysis.

[46] Wu X., Yang S.T., Wang H., Wang L., Hu W., Cao A., Liu Y. (2010). Influences of the size and hydroxyl number of fullerenes/fullerenols on their interactions with proteins. J. Nanosci. Nanotechnol..

[47] Araujo K.C., de M. B. Costa E.M., Pazini F., Valadares M.C., de Oliveira V. (2013). Bioconversion of quercetin and rutin and the cytotoxicity activities of the transformed products. Food Chem. Toxicol..

[48] Ning Y.N., Xiao B.L., Niu N.N., Moosavi-Movahedi A.A., Hong J. (2019). Glucose Oxidase Immobilized on a Functional Polymer Modified Glassy Carbon Electrode and Its Molecule Recognition of Glucose. Polymers.

[49] Al-Qurain A.A., Williams D.B., Mackenzie L., Roberts M.S., Wiese M.D. (2021). Simultaneous LC-MS/MS quantification of oxycodone, tramadol and fentanyl and their metabolites (noroxycodone, oxymorphone, O-desmethyltramadol, N-desmethyltramadol, and norfentanyl) in human plasma and whole blood collected via venepuncture and volumetric absorptive micro sampling. J. Pharm. Biomed. Anal..

[50] Karabiberoğlu Ş.U., Koçak Ç.C., Dursun Z. (2019). An over-oxidized poly(Rutin) modified electrode for selective and sensitive determination of catechol and hydroquinone. J. Electroanal. Chem..

[51] Zhao W.-J., Xiao B.-L., Song X.-Y., Meng X., Ma X.-X., Li Y.-Y., Hong J., Moosavi-Movahedi A.A. (2022). A Highly Sensitive Electrochemical Sensor Based on β-cyclodextrin Functionalized Multi-Wall Carbon Nanotubes and Fe_3_O_4_ Nanoparticles for Rutin Detection. J. Electrochem, Soc..

[52] Yang X.-L., Zhang Y.-S., Yang T., Geng F.-Y., Li D., Xiao B.-L., Hong J., Moosavi-Movahedi A.A., Ghourchian H. (2015). A soft-template nanostructured peroxidase based on cytochrome c and sodium decyl sulfate and its electrochemical properties on hydroxyl fullerenes modified glassy carbon electrode. J. Iran. Chem. Soc..

[53] Laviron E. (1979). General expression of the linear potential sweep voltammogram in the case of diffusionless electrochemical systems. J. Electroanal. Chem..

[54] Sheng K., Zhang Q., Li L., Wang Y., Ye B. (2020). A new voltammetric sensor and its application in pharmaceutical analysis for rutin. J. Environ. Sci. Health A.

[55] Martins L.O., Durao P., Brissos V., Lindley P.F. (2015). Laccases of prokaryotic origin: Enzymes at the interface of protein science and protein technology. Cell. Mol. Life Sci..

[56] Taraba A., Szymczyk K. (2020). Quercetin and rutin mixtures with alcohols: Spectroscopic and conductometric studies. J. Mol. Struct..

[57] Swamy N.K., Mohana K.N.S., Hegde M.B., Madhusudana A.M., Rajitha K., Nayak S.R. (2021). Fabrication of graphene nanoribbon-based enzyme-free electrochemical sensor for the sensitive and selective analysis of rutin in tablets. J. Appl. Electronchem..

[58] Wang Y., Zhang B., Tang Y., Zhao F., Zeng B. (2021). Fabrication and application of a rutin electrochemical sensor based on rose-like AuNPs-MoS_2_-GN composite and molecularly imprinted chitosan. Microchem. J..

